# The Spectrum, Tendency and Predictive Value of *PIK3CA* Mutation in Chinese Colorectal Cancer Patients

**DOI:** 10.3389/fonc.2021.595675

**Published:** 2021-03-26

**Authors:** Xinhui Fu, Hanjie Lin, Xinjuan Fan, Yaxi Zhu, Chao Wang, Zhiting Chen, Xiaoli Tan, Jinglin Huang, Yacheng Cai, Yan Huang

**Affiliations:** ^1^ Department of Pathology, The Sixth Affiliated Hospital, Sun Yat-sen University, Guangzhou, China; ^2^ Guangdong Institute of Gastroenterology, The Sixth Affiliated Hospital, Sun Yat-sen University, Guangzhou, China; ^3^ Guangdong Provincial Key Laboratory of Colorectal and Pelvic Floor Diseases, The Sixth Affiliated Hospital, Sun Yat-sen University, Guangzhou, China

**Keywords:** colorectal cancer, mutation spectrum, nomogram, *PIK3CA*, the predictive value, HRM test, cetuximab

## Abstract

**Background:**

*PIK3CA* is a high-frequency mutation gene in colorectal cancer, while its prognostic value remains unclear. This study evaluated the mutation tendency, spectrum, prognosis power and predictive power in cetuximab treatment of *PIK3CA* in Chinese CRC cohort.

**Methods:**

The *PIK3CA* exon 9 and 20 status of 5763 CRC patients was detected with Sanger sequencing and a high-resolution melting test. Clinicopathological characteristics of 5733 patients were analyzed. Kaplan-Meier method and nomogram were used to evaluate the overall survival curve and disease recurrence, respectively.

**Results:**

Fifty-eight types of mutations in 13.4% (771/5733) of the patients were detected. From 2014 to 2018, the mutation rate of *PIK3CA* increased from 11.0% to 13.5%. At stage IV, exon 20 mutated patients suffered shorter overall survival time than wild-type patients (multivariate COX regression analysis, HR = 2.72, 95% CIs = 1.47-5.09; *p*-value = 0.012). At stage III, *PIK3CA* mutated patients were more likely to relapse (multivariate Logistic regression analysis, exon 9: OR = 2.54, 95% CI = 1.34-4.73, *p* = 0.003; exon 20: OR = 3.89, 95% CI = 1.66-9.10, *p* = 0.002). The concordance index of the nomogram for predicting the recurrence risk of stage III patients was 0.685. After cetuximab treatment, the median PFS of *PIK3CA* exon 9 wild-type patients (n = 9) and mutant patients (n = 5) did not reach a significant difference (3.6 months vs. 2.3 months, Log-rank test, *p*-value = 0.513).

**Conclusions:**

We found that *PIK3CA* mutation was an adverse predictive marker for the overall survival of stage IV patients and recurrence of stage III patients, respectively. Further more, we suggested that *PIK3CA* exon 9 mutations are not negative predictors of cetuximab treatment in *KRAS*, *NRAS*, and *BRAF* wild-type mCRC patients.

## Introduction

Colorectal cancer (CRC) is the third most prevalent malignancy worldwide, which leads to more than 860,000 deaths every year (1). In China, CRC has become the third most common cancer and the fifth leading cause of cancer-related mortality (2), while the incidence is predicted to be growing (3).

To date, the most effective treatment of CRC is adjuvant chemotherapy after surgical resection. Recently, the significant predictive value of some genetic mutation status has been reported by various clinical studies. *KRAS* mutation status was proved to be a robust predictive biomarker for the efficacy of anti-epidermal growth factor receptor (anti-EGFR) therapies (4). *BRAF* mutation has been wildly recognized as a reliable indicator of poor prognosis (5, 6). *PIK3CA* is one of the most frequently mutated oncogenes in CRC. It was reported that about 15-20% of CRC patients carried *PIK3CA* mutation (7), 80% of which was found in exon 9 and exon 20 (8). Previous studies indicated that patients with *PIK3CA* mutation could benefit from regular Aspirin treatment (9, 10), while the prognostic impact of *PIK3CA* mutation has far been controversial (11–15). Besides, the relationship between *PIK3CA* mutant mCRC tumor and resistance of anti-EGFR agents, cetuximab, is lack of investigation and did not reach consistency (16–20).

Patients at stage III were recommended to receive chemotherapy treatments, and most of the chemotherapy regimens were 5-fluorouracil based, such as FOLFOX and XELOX (6). Some clinical studies reported that a large group of patients was found to show chemotherapy resistance (11–23). The predictive effect of *PIK3CA* mutations in chemotherapy regimens in CRC was rarely reported and remained unclear (24).

In this retrospective study, we analyzed *PIK3CA* mutations in 5763 CRC patients, using Sanger sequencing and high-resolution melting (HRM) test, and evaluated the associations between *PIK3CA* mutations and the clinicopathological characteristics. We also conducted a survival analysis to investigate the prognostic value of *PIK3CA* mutations, and evaluated the role of *PIK3CA* mutations in first-line chemotherapy. A nomogram was then constructed to predict the recurrence risk of CRC patients at stage III. Further, the predictive effect of *PIK3CA* exon 9 mutation in cetuximab treatment was investigated with wild-type *KRAS*, *NRAS*, *BRAF* mCRC patients.

## Materials and Methods

### Subjects

This study was approved by the Ethics Committee of the Sixth Affiliated Hospital of Sun Yat-sen University (L2017ZSLYEC-003). All patients underwent an informed consent process approved by the Hospital Institutional Review Board.

Our study included 5763 CRC patients diagnosed at the Sixth Affiliated Hospital of Sun Yat-sen University from January 2014 to December 2018. In this study, colorectal tumor specimens were fixed in formalin, embedded in paraffin after surgery, and confirmed histologically. The clinicopathologic features of these patients were collected from their medical records. The definition of the rectum is 12 cm or less from the anal verge.

Among the 5763 CRC patients, a sub-cohort composed of 1946 patients with available follow-up records was used to evaluate the prognosis value of *PIK3CA* exon 9 and 20 mutations. Another sub-cohort composed of 377 stage III patients who had received at least six courses of 5-fluorouracil based chemotherapy treatment after surgical excision was used to evaluate the associations between *PIK3CA* mutations and CRC recurrence.

### Mutation Detections

In this study, 5763 patients’ primary intestinal tumors were collected for genetic testing. Genomic DNA extraction and Sanger sequencing were followed by the procedure of our previous study (25).

Besides, high-resolution melting (HRM) test was also applied to detect the mutation status of *PIK3CA*. In brief, the PCR reagent used in the HRM test was LightCycler 480 High-Resolution Melting Master Reagent Kit (Cat no: 04909631001, Roche Diagnostics Indianapolis, USA). The reaction system of the HRM test was: 50-100ng genomic DNA, 10μl 2×Master mix, 2.8μl MgCl_2_, and 1μl 10µM of each primer for exon 9 test, or 0.8μl 10µM of each primer for exon 20 test, and the mixtures were added up to 15μl with H_2_O. The primers of the exon 9 HRM test were: 5’-GAGACAATGAATTAAGGGAAAATG-3’ and 5’-CACTTACCTGTGACTCCATAG-3’; The primers of the exon 20 HRM test were: 5’-ACCCTAGCCTTAGATAAAACTGAGC-3’ and 5’-TCCATTTTTGTTGTCCAGCCACCAT-3’. The reaction conditions of cycling and melting for exon 9 HRM test were: 95°C for 10 min; 45 cycles of 95°C for 20s, 60°C for 20s and 72°C for 20s; 95°C; for 30s and 37°C for 30s; followed by a high-resolution melt of 74°C to 87°C with 45 acquisitions/°C 40°C for 10s. The exon 20 HRM test’s reaction condition was the same as that of exon 9 except that the annealing temperature was 62°C.

The *PIK3CA* exon 9 and 20 HRM tests’ detection limits were evaluated with spiked plasmid samples containing wild-type copies and mutant-type copies at various percentages. In detail, the exon 9 mutation type was c.1633G>A, and the initial concentration of mutant and wild-type plasmids were both 9.2×10^5^ copies/μl. The exon 20 mutation type was c.3140A>T, and the initial concentration of mutant and wild-type plasmids were both 4.9×10^5^ copies/μl. *PIK3CA* mutant plasmid was mixed with its corresponding wild type plasmid at various dilutions, at ratio of 50%, 40%, 30%, 20%, 10%, 8%, 6%, 5%. The minimum detectable dilution serves as the detection limit in this study.

Sanger sequencing was wildly considered a golden standard method for mutation detection. According to the Sanger sequencing results, sensitivities and specificities were calculated for *PIK3CA* exon 9 and 20 by HRM tests. Only the samples that obtained consistent results from the two methods were included for this study’s subsequent analysis.

For association analysis, the mutation information of *KRAS* exon 2 and *BRAF* codon 600 in our cohort was also obtained by the previously described detection method (26).

### Statistic Analysis

Chi-square test, Mann-Whitney U test, and Fisher’s exact test were applied to analyze the association of *PIK3CA* exon 9 and 20 mutation status with clinical characteristics, including the mutation status of *KRAS* exon 2 and *BRAF^V600E^*. The analyses were initially evaluated with continuous variables, categories data analysis, and further accessed with logistic regression models to evaluate the association based on estimating the odds ratios (OR) and their 95% confidence intervals (CIs). The significance test was two-sided, and a *p*-value < 0.05 was considered as statistically significant. All the statistical analyses were performed with SPSS 20.0 packages (SPSS, Chicago, IL, USA).

The annual mutation rates of *PIK3CA* exon 9 and 20 from 2014 to 2018 were calculated separately, and the mutation rates tendency was analyzed with the joinpoint regression model (Joinpoint 4.6.0.0., Calverton, MD, USA).

Kaplan-Meier survival curves for overall survival (OS) of 1946 patients with available follow-up records were performed with GraphPad Prism 5 (Graph Pad Software Inc., San Diego, CA, USA). The Log-rank test was used to assess the significance, and the *p*-value less than 0.05 was considered statistically significant. Besides, the logistic regression was used to screen other clinical characteristics related to the recurrence of patients at stage III. These variables were then enrolled to create a nomogram with rms package (version 5.1-4, https://cran.r-project.org/web/packages/rms/) in R statistical software (version 3.4.3).

## Results

### Demographic and Clinical Characteristics

**Figure 1:**
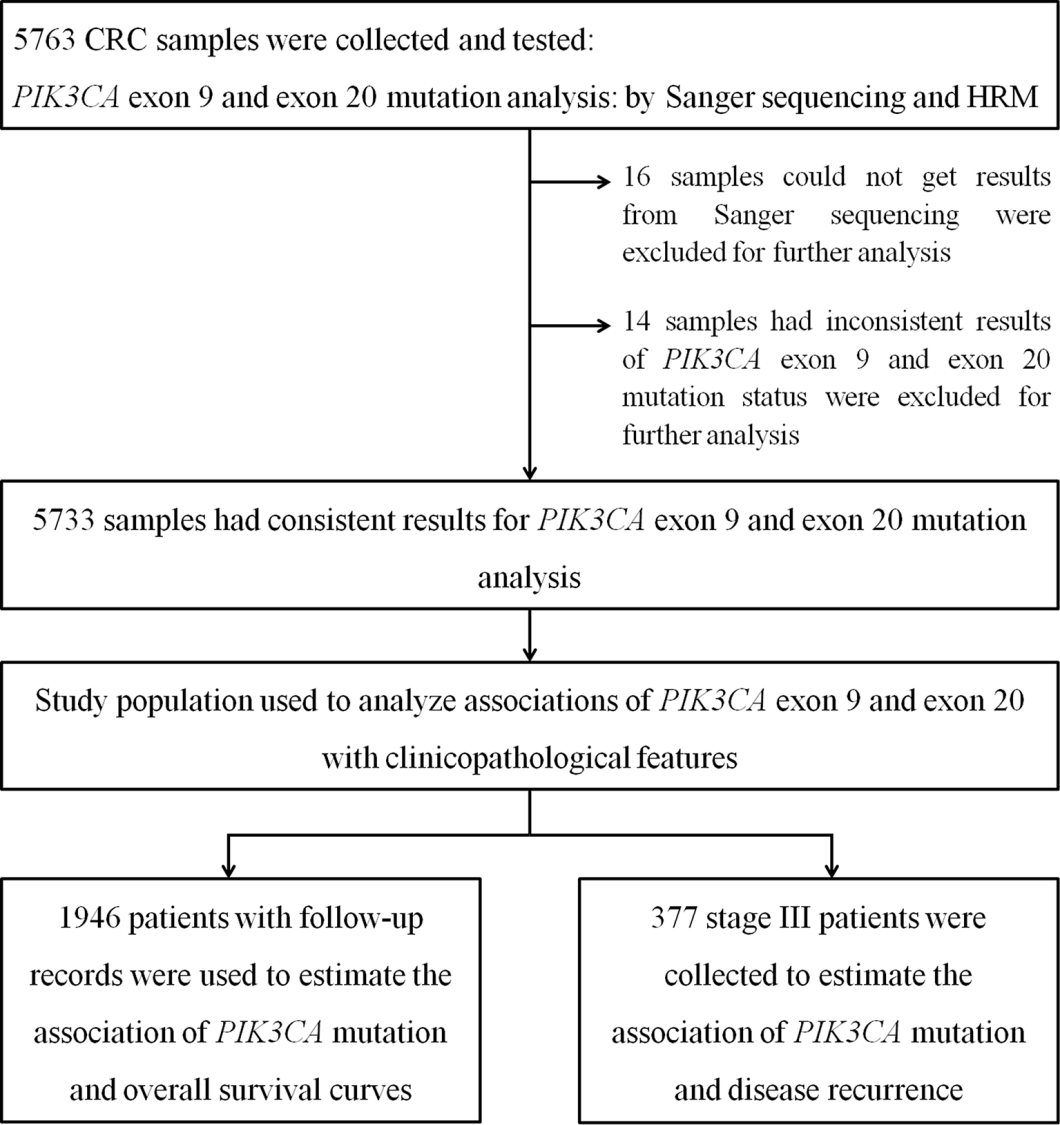
Selection of study population.

The study cohort involved 5733 patients (**Figure 1**), getting the general profiles of CRC patients in China. Some basic clinical features of the cohort were summarized in **Table 1**. Briefly, there were more male patients (60.9%) in this cohort. The age of the first diagnosis ranged from 17 to 96 years old. We assigned patients into four groups, younger than 45 years old, between 45 to 49 years old, between 50 to 75 years old, and older than 75 years old, with 13.8%, 9.4%, 67.2%, and 9.6% of the study cohort respectively. For all CRC patients’ tumor sites, 47.4% of tumors were located in the rectum, 30.9% of tumors were located in the left colon, and 21.6% of tumors were located in the right colon. The vast majority of patients included in this study were tubular adenocarcinoma, of which 17.6%, 74.6%, and 7.8% were well, moderately, and poorly differentiated, respectively. Among these 5733 patients, only 3153 patients’ TNM stages information was available, in which 10.9%, 37.6%, 34.9%, and 16.5% of patients were assigned to stage I, stage II, stage III, and stage IV.

**Table 1:** Associations of *PIK3CA* exon 9 and 20 mutation status with clinicopathologic characteristics.

Characteristics	No. of patients(n = 5733)	Mutant *PIK3CA* exon 9 (n = 511)	Wild-type *PIK3CA* exon 9 (n = 5222)	*p*	Mutant *PIK3CA* exon 20 (n = 270)	Wild-type *PIK3CA* exon 20 (n = 5463)	*p*
Gender				0.123			0.335
Male	3492 (60.9%)	295 (8.4%)	3197 (91.6%)		172 (4.9%)	3320 (95.1%)	
Female	2241 (39.1%)	216 (9.6%)	2025 (90.4%)		98 (4.4%)	2143 (95.6%)	
Age, years				0.453^1^			0.001^1^
Mean(SD)	58.8 (12.9)	59.1 (13.7)	58.7 (12.9)		55.9 (13.8)	58.9 (12.9)	
Median	60.0	60.0	60.0		58.0	60.0	
Range	17.0-96.0	23.0-95.0	17.0-96.0		18.0-89.0	17.0-96.0	
Age, years				0.084			<0.001
<45	794 (13.8%)	79 (9.9%)	715 (90.1%)		57 (7.2%)	737 (92.8%)	
45-49	539 (9.4%)	39 (7.2%)	500 (92.8%)		33 (6.1%)	506 (93.9%)	
50-75	3852 (67.2%)	332 (8.6%)	3520 (91.4%)		159 (4.1%)	3693 (95.9%)	
>75	548 (9.6%)	61 (11.1%)	487 (88.9%)		21 (3.8%)	527 (96.2%)	
Tumor site				<0.001			<0.001
Rectum	2717 (47.4%)	184 (6.8%)	2533 (93.2%)		96 (3.5%)	2621 (96.5%)	
Left colon*	1773 (30.9%)	161 (9.1%)	1612 (90.9%)		62 (3.5%)	1711 (96.5%)	
Right colon*	1241 (21.6%)	166 (13.4%)	1075 (86.6%)		112 (9.0%)	1129 (91.0%)	
Differentiation of tubular adenocarcinoma				0.012			0.902
Well	932 (17.6%)	89 (9.5%)	843 (90.5%)		40 (4.3%)	892 (95.7%)	
Moderate	3954 (74.6%)	352 (8.9%)	3602 (91.1%)		180 (4.6%)	3774 (95.4%)	
Poor	415 (7.8%)	20 (4.8%)	395 (95.2%)		20 (4.8%)	395 (95.2%)	
Nontubular adenocarcinoma	432	50	382		30	402	
*PIK3CA* exon 9 status				–			0.002
Wild type	5222 (91.1%)				260 (5.0%)	4962 (95.0%)	
Mutant	511 (8.9%)				10 (2.0%)	501 (98.0%)	
*PIK3CA* exon 20 status				0.002			–
Wild type	5463 (95.3%)	501 (9.2%)	4962 (90.8%)				
Mutant	270 (4.7%)	10 (3.7%)	260 (96.3%)				
*KRAS* exon 2 status				<0.001			<0.001
Wild type	3459 (60.3%)	184 (5.3%)	3275 (94.7%)		131 (3.8%)	3328 (96.2%)	
Mutant	2274 (39.7%)	327 (14.4%)	1947 (85.6%)		139(6.1%)	2135 (93.9%)	
*BRAF^V600E^* status				1.000			0.365
Wild type	5555 (96.9%)	495 (8.9%)	5060 (91.1%)		259 (4.7%)	5296 (95.3%)	
Mutant	178 (3.1%)	16 (9.0%)	162 (91.0%)		11 (6.2%)	167 (93.8%)	

*Left colon: descending colon, sigmoid colon, and rectosigmoid; Right colon: cecum, ascending colon and transverse colon.

Spearman Chi-square test.

^1^Mann-Whitney U test.

### Associations of *PIK3CA* Exon 9 and 20 Mutations With Clinicopathologic Characteristics

The associations of *PIK3CA* exon 9 and 20 mutations and clinicopathologic characteristics were shown in **Table 1** and **Table S1**. In exon 9, mutations were more likely to occur at right colon (13.4%) than at other sides (6.8% at rectum, 9.1% at left side; *p*-value < 0.001), while less likely to present at poor differentiation tumors (4.8%) than at well and moderate differentiation tumors (9.5% and 8.9% respectively; *p*-value = 0.012). In TNM stage analysis, the exon 9 mutation frequency was higher in stage II (18.3%) than the other three stages (12.8% at stage I, 12.4% at stage III, and 15.5% at stage IV, *p*-value = 0.001, **Table S1**). In exon 20, mutations were more likely to occur in younger patients (7.2% in patients younger than 45 years old and 6.1% in patients between 45 to 49 years old) than those older than 50 years old (4.1% in patients between 50 to 75 years old and 3.8% at older than 75 years old, *p*-value < 0.001), as well as more likely to occur at the right colon (9.0%) than other sites (both 3.5% at the rectum and left colon, *p*-value < 0.001). In TNM stage analysis, exon 20 mutations were significantly more common at stage II (11.4%) than in other stages (5.5% at stage I, 5.9% at stage III, and 6.5% at stage IV; *p*-value < 0.001, **Table S1**).

Among all the 5733 patients, only ten patients mutated in both exons simultaneously, which indicated that exon 9 and exon 20 of *PIK3CA* are mutually exclusive in CRC (**Table 1**). *KRAS* exon 2 mutations were associated with *PIK3CA* exon 9 and exon 20 mutations (*p*-value < 0.001 for both exons), while *BRAF^V600E^* mutation did not show any association with both *PIK3CA* exons.

Features which were significantly associated with *PIK3CA* mutations in **Table 1** or considered to have important effects in clinical practice were involved into logistic regression analysis, and the results were shown in **Table S2**. In both exons, mutations were more likely to present at right colon (in exon 9, OR = 1.86, 95% CIs = 1.53-2.26; in exon 20, OR = 2.72, 95% CIs = 2.12-3.50; *p*-value < 0.001 for both exons), as well as in stage II (in exon 9, OR= 1.46, 95% CIs = 1.20-1.78; in exon 20, OR = 2.01, 95% CIs = 1.55-2.60; *p*-value < 0.001 for both exons). In exon 9, mutations were less likely detected in poor differentiated tumor (OR = 0.51; 95% CIs = 0.32-0.81, *p*-value = 0.004). Higher *PIK3CA* exon 20 mutation frequency was associated with patients younger than 50 years old (OR = 1.70, 95% CIs = 1.31-2.21, *p*-value < 0.001).

### The Trends of the Mutation Rate of *PIK3CA* Mutations

The mutation rates of *PIK3CA* among Chinese CRC patients from 2014 to 2018 were shown in **Figure S1** and **Table S3**. The mutation rate of *PIK3CA* exon 9 shows a slightly ascending tendency from 6.2% in 2014 to 8.9% in 2018 (**Figure S1A**. APC = 7.95, *p*-value > 0.05). The mutation rate of *PIK3CA* exon 20 remained nearly stable, as 5.00% in 2014 and 4.7% in 2018, (**Figure S1B**. APC = 0.30, *p*-value > 0.05). Combined both exons, the mutation rate of *PIK3CA* shows a gradually increasing tendency from 11.0% in 2014 to 13.5% in 2018 (**Figure S1C**. APC = 5.08, *p*-value > 0.05).

### Mutation Types and Classification of *PIK3CA* Gene in CRC Patients

*PIK3CA* exon 9 and 20 mutations were tested in all 5763 samples with Sanger sequencing and the HRM test (**Figure 1**). Sanger sequencing failed to generate analyzable results from 16 samples, which can be detected by the HRM test. In the rest of 5747 samples, 14 had inconsistent results. The detection limits of *PIK3CA* exon 9 and exon 20 HRM tests were both 5% (**Supplementary Figures S2** and **S3**), which is more sensitive than Sanger sequencing (20%). The specificities of *PIK3CA* exon 9 and exon 20 HRM tests were 99.77% and 99.96%, respectively, and the sensitivities of these two detecting methods were both 100% (Chinese patent, NO. 201610524420.9).

Among the final cohort with 5733 samples, 13.4% (771/5733) patients mutated in *PIK3CA* with 58 types of mutations (**Table 2**). Among these 771 mutated patients, 501 mutated at exon 9, 260 mutated at exon 20, and only 10 mutated at both exons simultaneously.

**Table 2:** Mutation types and classification in *PIK3CA* exon 9 and 20 of Chinese CRC patients.

Mutation type	Amino acid change	Frequency in mutated patients	Cosimc ID	Detected by ARMS-based kits	Variants classification*	Reference for classification(PMID)
c.1633G>A	E545K	32.0% (247/771)	COSM763	Yes	II	32259783 (27); 31091374 (28);
c.3140A>G	H1047R	23.6% (182/771)	COSM775	Yes	I	20619739 (16);
c.1624G>A	E542K	17.6% (136/771)	COSM760	Yes	II	32259783 (27); 31091374 (28);
c.3140A>T	H1047L	3.9% (30/771)	COSM36289	Yes	II	32259783 (27); 31091374 (28);
c.1636C>A	Q546K	3.5% (27/771)	COSM776	No	II	17376864 (29); 29985963 (30); 29970892 (24);
c.1634A>G	E545G	2.5% (19/771)	COSM764	No	II	32259783 (27); 31091374 (28);
c.1637A>G	Q546R	1.8% (14/771)	COSM12459	No	II	29970892 (24); 29533785 (31); 26627007 (32);
c.1634A>C	E545A	1.8% (14/771)	COSM12458	No	II	32259783 (27); 31091374 (28);
c.3129G>T	M1043I	1.7% (13/771)	COSM773	No	II	17376864 (29); 29970892 (24); 15930273 (33); 22430209 (34);
c.3139C>T	H1047Y	1.0% (8/771)	COSM774	No	II	32259783 (27); 31091374 (28);
c.1636C>G	Q546E	0.9% (7/771)	COSM6147	No	II	29970892 (24); 26627007 (32);
c.1637A>C	Q546P	0.6% (5/771)	COSM767	No	II	17376864 (29); 29970892 (24); 26627007 (32);
c.3145G>C	G1049R	0.6% (5/771)	COSM12597	No	II	29970892 (24); 26627007 (32);
c.3129G>A	M1043I	0.5% (4/771)	COSM29313	No	II	17376864 (29); 29970892 (24); 15930273 (33); 22430209 (34);
c.1635G>C	E545D	0.5% (4/771)	COSM27374	No	II	32259783 (27); 31091374 (28);
c.1637A>T	Q546L	0.5% (4/771)	COSM25041	No	III	Literature not found
c.1638G>T	Q546H	0.5% (4/771)	COSM24712	No	III	Literature not found
c.1635G>T	E545D	0.4% (3/771)	COSM765	Yes	II	32259783 (27); 31091374 (28);
c.1625A>T	E542V	0.3% (2/771)	COSM762	No	II	29970892 (24); 26627007 (32);
c.[1633G>A;3140A>G]	E545K; H1047R	0.3% (2/771)	COSM763; COSM775	Yes	I	32259783 (27); 31091374 (28); 20619739 (16);
c.3075C>T	T1025T	0.3% (2/771)	COSM21451	No	IV	26627007 (32);
c.3127A>G	M1043V	0.3% (2/771)	COSM12591	No	II	17376864 (29); 29970892 (24); 18097548 (35);
c.3141T>A	H1047Q	0.3% (2/771)	COSM1041524	No	III	Literature not found
c.1601C>A	S534Y	0.1% (1/771)	Not included	No	III	Literature not found
c.1613A>T	D538V	0.1% (1/771)	Not included	No	III	Literature not found
c.1616C>G	P539R	0.1% (1/771)	COSM759	No	II	17376864 (29); 29970892 (24); 18951408 (36);
c.[1620C>A;3129G>T]	L540L; M1043I	0.1% (1/771)	Not included; COSM773	No	II	25146167 (37);
c.1622C>T	S541F	0.1% (1/771)	COSM6438100	No	III	Literature not found
c.1625A>G	E542G	0.1% (1/771)	COSM761	No	III	Literature not found
c.[1624G>A;3127A>T]	E542K; M1043L	0.1% (1/771)	COSM760; COSM5731063	Yes	II	32259783 (27); 31091374 (28); 29970892 (24); 31699932 (38); 29533785 (31);
c.[1624G>A;3139C>T]	E542K; H1047Y	0.1% (1/771)	COSM760; COSM774	Yes	II	32259783 (27); 31091374 (28);
c.1631C>A	T544N	0.1% (1/771)	COSM249872	No	III	Literature not found
c.1631C>T	T544I	0.1% (1/771)	COSM249876	No	III	Literature not found
c.1633G>C	E545Q	0.1% (1/771)	COSM27133	No	II	32259783 (27); 31091374 (28);
c.1635G>A	E545E	0.1% (1/771)	COSM1716554	No	IV	26627007 (32); 31404155 (39);
c.[1635G>A;1636C>A]	E545E; Q546K	0.1% (1/771)	COSM1716554; COSM776	No	II	32259783 (27); 31091374 (28); 17376864 (29); 29985963 (30); 29970892 (24);
c.[1633G>A;3129G>A]	E545K; M1043I	0.1% (1/771)	COSM763; COSM29313	Yes	II	32259783 (27); 31091374 (28);
c.[1633G>A;3139C>T]	E545K; H1047Y	0.1% (1/771)	COSM763; COSM774	Yes	II	32259783 (27); 31091374 (28);
c.[1634A>G;3139C>T]	E545G; H1047Y	0.1% (1/771)	COSM764; COSM774	No	II	32259783 (27); 31091374 (28);
c.[1633G>A;3143A>G]	E545K; H1048R	0.1% (1/771)	COSM763; COSM36289	Yes	II	32259783 (27); 31091374 (28);
c.[1637A>G;3140A>G]	Q546R; H1047R	0.1% (1/771)	COSM775; COSM775	Yes	I	20619739 (16); 29970892 (24); 29533785 (31); 26627007 (32);
c.1637_1638delinsGT	Q546R	0.1% (1/771)	Not included	No	II	29970892 (24); 29533785 (31); 26627007 (32);
c.1637_1638delinsCC	Q546P	0.1% (1/771)	COSM6959028	No	II	17376864 (29); 29970892 (24); 26627007 (32);
c.1639G>C	E547Q	0.1% (1/771)	Not included	No	III	Literature not found
c.1645G>T	D549Y	0.1% (1/771)	Not included	No	III	Literature not found
c.1651C>T	L551L	0.1% (1/771)	COSM308546	No	III	22287190 (40);
c.3073A>G	T1025A	0.1% (1/771)	COSM771	No	II	17376864 (29); 16764926 (41);
c.3106G>A	E1036K	0.1% (1/771)	Not included	No	III	Literature not found
c.3113A>G	Y1038C	0.1% (1/771)	COSM27489	No	III	Literature not found
c.3135T>C	D1045D	0.1% (1/771)	Not included	No	III	Literature not found
c.[3133G>A;3140A>G]	D1045N; H1047R	0.1% (1/771)	COSM775; COSM775	No	I	20619739 (16);
c.3139C>G	H1047D	0.1% (1/771)	Not included	No	III	Literature not found
c.3145G>A	G1049S	0.1% (1/771)	COSM777	No	III	Literature not found
c.3146G>C	G1049A	0.1% (1/771)	COSM27158	No	III	Literature not found
c.3218G>A	*1073*	0.1% (1/771)	Not included	No	IV	Literature not found
c.3165dup	M1055fs	0.1% (1/771)	Not included	No	III	30941989 (42); 28461758 (43); 21990951 (44);
c.3126dup	Q1042fs	0.1% (1/771)	Not included	No	III	30941989 (42); 28461758 (43); 21990951 (44);
c.3204dup	N1068fs	0.1% (1/771)	Not included	No	III	30941989 (42); 28461758 (43); 21990951 (44);

Variants classification*: according to the instruction guidelines of ACMG (45). II: variants of potential clinical significance; III: variants of unknown clinical significance; IV: benign or likely benign variants.

Among 58 mutation types, the 10 most frequent mutation types were: E545K (32.0%), H1047R (23.6%), E542K (17.6%), H1047L (3.9%), Q546K (3.5%), E545G (2.5%), Q546R (1.8%), E545A (1.8%), M1043I (1.7%) and H1047Y (1.0%). However, the other 48 types of mutations were only detected in 1.4% (81/5733) of CRC patients, which represented 10.5% (81/771) of *PIK3CA* mutated patients.

Among 58 mutation types, only 12 types of mutations could be detected by ARMS-based commercial *PIK3CA* mutation detection kits, which comprised 78.6% (606/771) of mutated patients.

In this study, we also annotated these somatic mutations following the instruction of ACMG guides on the interpretation of sequence variants (45). There were 4, 30, 21, and 3 mutation types in *PIK3CA* that were classified as Tier I, II, III, and IV variants in colorectal cancer, respectively (**Table 2**).

### Survival Analysis

**Figure 2:**
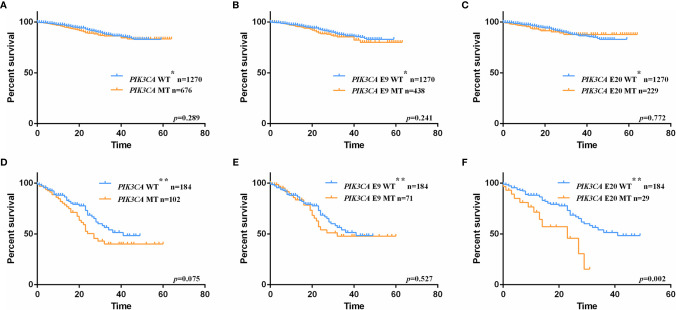
Kaplan-Meier plots of overall survival (OS) for CRC patients with and without *PIK3CA* mutation. **(A)** at all stage, patients with *PIK3CA* mutation *vs.* patients with wild-type allele, *p*-value = 0.289; **(B)** at all stage, patients with exon 9 mutation *vs.* patients with wild-type allele, *p*-value = 0.241; **(C)**. at all stage, patients with exon 20 mutation *vs.* patients with wild-type allele, *p*-value = 0.772; **(D)** at stage IV, patients with *PIK3CA* mutation *vs.* patients with wild-type allele, *p*-value = 0.075; **(E)** at stage IV, patients with exon 9 mutation *vs.* patients with wild-type allele, *p*-value = 0.527; **(F)** at stage IV, patients with exon 20 mutation *vs.* patients with wild-type allele, *p*-value = 0.002. WT* represents stage I-IV wild-type patients with neither exon 9 nor exon 20 mutations. WT** represents stage IV wild-type patients with neither exon 9 nor exon 20 mutations.

In order to evaluate the prognosis value of *PIK3CA* mutation in CRC patients, 1946 patients with available follow-up information were collected for survival analysis (clinicopathologic characteristics were shown in **Table S4**). The follow-up started from the day of surgery and ended on August 30, 2019. The median follow-up for the 1946 cohort is 16 months (0-64 months). Their overall survival rates were analyzed with the Kaplan-Meier method. In this follow-up cohort, 447 patients carried exon 9 mutations, 238 patients carried exon 20 mutations, and the remaining 1270 patients had wild-type *PIK3CA* gene. During the following-up, 178 patients died, among which 138 patients died from colorectal cancer or related diseases. No significant difference was detected between patients with and without *PIK3CA* mutation (Log-rank test, *p*-value = 0.289; **Figure 2A**), exon 9 mutation (Log-rank test, *p*-value = 0.241; **Figure 2B**), and exon 20 mutation (Log-rank test, *p*-value = 0.772; **Figure 2C**), respectively. For further analyses, patients were divided into four subgroups according to their TNM stages at the first diagnosis and conducted the survival analysis separately (**Figures 2D–F**). We found that only patients at stage IV who carried exon 20 mutations experienced significantly shorter OS than wild-type patients (median follow-up: 14 months; median survival for *PIK3CA* wild-typed and exon 20 mutated patients: 40 months *vs.* 23 months; Log-rank test, *p*-value = 0.002; **Figure 2F**). To assess the *PIK3CA* exon 20 mutations’ influence on the survival of stage IV patients, the COX regression model was applied. In comparison with patients carried *PIK3CA* exon 20 wild-type tumors, those with *PIK3CA* exon 20 mutated stage IV patients showed a decrease in OS (univariate HR = 2.52, 95% CIs = 1.38-4.59; *p*-value = 0.003, **Table 3**). In the multivariate COX regression model, *PIK3CA* exon 20 mutation was associated with a significant decrease in OS of stage IV CRC patients (HR = 2.72, 95% CIs = 1.47-5.09; *p*-value =0.012, **Table 3**).

**Table 3:** Cox regression model associations between clinicopathologic characteristics and mortality in stage IV CRC patients.

Variables	Univariate analysis		Multivariate analysis	
	HR (95% CIs)	*p*	HR (95% CIs)	*p*
*PIK3CA* exon 20 mutation (*vs.* wild-type)	2.52 (1.38-4.59)	0.003	2.19 (1.19-4.03)	0.012
Right colon (*vs.* others)	2.22 (1.37-3.60)	0.001	2.40 (1.47-3.94)	0.001
Poor differentiation (*vs.* others)	2.47 (1.49-4.11)	<0.001	2.39 (1.42-4.02)	0.001
Age > 75 years (*vs.* others)	2.13 (1.22-3.73)	0.008	2.15 (1.21-3.80)	0.009

CIs, confidence intervals; HR, hazard ratio.

### Recurrence Nomogram of Patients With Stage III CRC

In this study, the impact of *PIK3CA* mutations on 5-fluorouracil based chemotherapy treatment was evaluated in stage III patients who had received consistent chemotherapy in our hospital (clinicopathologic characteristics were shown in **Table S5**). In total, 377 patients were included in this sub-cohort, and 76 (20.2%) were observed to have disease progression (median follow-up: 13 months; median time-to relapse: 13 months). Univariate and multivariate logistic analyses were applied to screen independent factors relative to disease recurrence. In univariate and multivariate analysis, *PIK3CA* exon 9, and tumor sites were related to stage III patients’ disease relapse (*p*-value < 0.05 for all, **Table 4**), indicating that these variants were independent predictive markers in recurrence. Besides, *PIK3CA* exon 20 was found to be on the cusp of conventional statistical significance (OR = 2.15, 95% CIs = 0.99-4.65, *p*-value = 0.053) in univariate analysis. In multivariate analysis, it showed significantly associated with recurrence in stage III patients (OR = 3.89, 95%CIs = 1.66-9.10, *p*-value = 0.002). The same situation also occurred in patients’ gender and tumor differentiation (**Table 4**).

**Table 4:** Univariate and multivariate analyses of disease recurrence at stage III CRC patients.

Variables	Univariate analysis		Multivariate analysis	
	OR (95% CIs)	*p*	OR (95% CIs)	*p*
*PIK3CA* exon 9 mutation (*vs.* wild-type)	2.26 (1.26-4.06)	0.007	2.54 (1.37-4.73)	0.003
*PIK3CA* exon 20 mutation (*vs.* wild-type)	2.15 (0.99-4.65)	0.053	3.89 (1.66-9.10)	0.002
Female (*vs.* male)	0.69 (0.42-1.14)	0.149	0.56 (0.33-0.97)	0.038
Right colon (*vs.* others)	0.43 (0.22-0.84)	0.013	0.38 (0.19-0.76)	0.006
Well differentiation (*vs.* others)	0.29 (0.07-1.23)	0.093	0.20 (0.04-0.89)	0.035

CIs, confidence intervals; OR, odds ratio.

**Figure 3:**
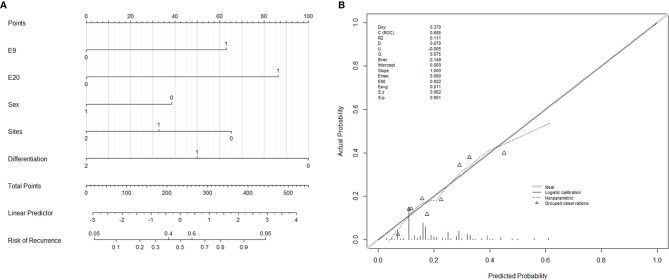
Nomogram and calibration curves for predicting the probability of disease recurrence in Stage III CRC patients. **(A)** E9: *PIK3CA* exon 9, 0 = wild-type, 1 = mutation; E20: *PIK3CA* exon 20, 0 = wild-type, 1 = mutation; Sex: 0 = female, 1 = male; Sites: tumor sites, 0 = rectum, 1 = left colon, 2 = right colon; Differentiation: tubular adenocarcinoma differentiation, 0 = poor differentiation, 1 = moderate differentiation, 2 = well differentiation. **(B)** Calibrate curve of nomogram. The C-index of this nomogram is 0.685.

A nomogram incorporated these five variables of stage III patients’ disease recurrence was established (**Figure 3A**), and the concordance index of this nomogram was 0.685, which serves as a reasonable accuracy for prediction. The calibration curves also showed high coherence between the observed and predicted disease relapse in the nomogram (**Figure 3B**).

### *PIK3CA* Exon 9 Mutations Is Not A Negative Biomarker for Wild-Type *KRAS, NRAS*, and *BRAF* mCRC Patients in Cetuximab Treatment

We collected a cohort consist of 14 stage IV patients who received cetuximab treatment (**Table 5**) from the 5733 patients. All of these 14 patients carried wild-typed *KRAS*, *NRAS*, and *BRAF*. Five patients carried *PIK3CA* exon 9 mutations, and the rest nine patients carried wild-typed *PIK3CA*. For wild-typed *PIK3CA* patients, most of them (7/9) were evaluated as stable disease (SD), one patient was evaluated as partial response (PR), and one patient was evaluated as progressive disease (PD). For *PIK3CA* exon 9 mutant patients, four patients were evaluated as SD, and one was evaluated as PR. The disease control rate (DCR) was 88.9% (8/9) in wild-typed patients and 100% (5/5) in mutant patients. The progression-free survival (PFS) time ranged from 1.2 to 9.4 months in wild-type patients and from 1.3 to 12.2 months in mutant patients. The median PFS of the two groups did not reach a significant difference (3.6 months vs. 2.3 months, Log-rank test, *p*-value =0.513).

**Table 5:** Cetuximab response in 14 wild-type *KRAS*, *NRAS* and *BRAF* mCRC patients with/without *PIK3CA* exon 9 mutations.

Patient	Age	Gender	*PIK3CA* exon 9 mutation	Tumor location	Tumor differentiation	Before or after resection	Courses of cetuximab	PFS (m)	Clinical response
A	60	Female	E545K	Right colon	Medium	Before	9	5.3	SD
B	50	Female	E545K	Left colon	Poor	Before	17	12.2	SD
C	52	Male	E542K	Left colon	Moderate	After	5	2.3	SD
D	64	Male	E542K	Left colon	Moderate	After	3	1.3	PR
E	63	Male	E542K	Left colon	Moderate	After	4	1.5	SD
F	53	Male	Wild-type	Left colon	Moderate	After	16	9.4	SD
G	32	Male	Wild-type	Left colon	Moderate	Before	2	1.3	SD
H	70	Male	Wild-type	Left colon	Moderate	Before	8	3.5	SD
I	39	Male	Wild-type	Left colon	Well	Before	7	3.0	PR
J	57	Male	Wild-type	Left colon	Moderate	After	3	1.2	SD
K	53	Female	Wild-type	Left colon	Well	Before	8	5.6	SD
L	45	Male	Wild-type	Left colon	Moderate	Before	8	3.6	SD
M	53	Male	Wild-type	Left colon	Moderate	After	3	2.0	PD
N	62	Male	Wild-type	Left colon	Poor	After	11	7.1	SD

PFS, progression-free survival; SD, stable disease; PR, partial response; PD, progressive disease.

## Discussion

In this study, we reported 58 types of *PIK3CA* mutation in Chinese CRC patients with two detecting methods. Most of the commercial kits for *PIK3CA* mutation detection were based on the ARMS-PCR technique, which can only detect five types of *PIK3CA* mutations (E542K, E545K, E545D, H1047L, and H1047R). In this case, there were only 12 mutation types in our real-world data that could be detected by such commercial kits, while 21.4% of mutated patients will be missed. Since we found that *PIK3CA* mutation was a valuable predictive biomarker in survival and disease recurrence, it is essential to achieve precise results in clinical testing. Here we proved that the HRM test was a more sensitive detection method than Sanger sequencing at a lower cost, which can also cover all the 58 mutation types we detected. For these reasons, it can be used as an auxiliary detection method.

Moreover, our study indicated that *PIK3CA* mutation was a neutral biomarker when considered patients at all stages (n = 1946). However, *PIK3CA* exon 20 was an adverse prognostic factor for CRC patients at stage IV (n = 213). We also found that *PIK3CA* mutation was a potential molecular biomarker for predicting resistance to 5-fluorouracil based chemotherapy regimens in stage III CRC patients. These findings may provide instructive information in clinical practice.

Mutations at *PIK3CA* could activate the PI3K/AKT signaling pathway, which is an essential factor that leads to the occurrence of various human malignancies (46–48). High mutation frequency was observed in multiple cancers, including breast cancer, bladder cancer, and colorectal cancer (8). Previous researches reported that the mutation rates of *PIK3CA* in CRC are 10-20% (7). In this study, we found that about 13.4% of Chinese CRC patients carried *PIK3CA* mutation, 8.7% were at exon 9, 4.5% were at exon 20, and 0.2% were found at both exons. The mutation rates of *PIK3CA* in Chinese CRC patients were varied from different studies, which might be due to the size of the cohort and detection technique (49, 50). Our research collected a large cohort of Chinese CRC patients and detected *PIK3CA* mutations with two different methods, which ensured that the mutation rates reported in this study are representative.

Though the mutation rate was relatively high, the prognostic effect of *PIK3CA* in CRC remains controversial. Some studies showed that *PIK3CA* mutation was associated with a shorter OS. For example, Ogino et al. found that *PIK3CA* mutations were an adverse prognostic factor in CRC patients at stage I-III, while this effect was only restricted to patients with wild-type *KRAS* (11). Besides, some studies indicated that mutations at different exons carried different prognostic effects. Research conducted by Farina et al. found that patients with *PIK3CA* exon 20 mutations conferred a more reduced disease-free survival (DFS) than patients with wild-type *PIK3CA* in stage III, and such an adverse effect was not observed in patients with *PIK3CA* exon 9 mutations (13). More studies, however, found that *PIK3CA* is a neutral prognostic factor that did not show any prognostic effects in CRC patients (14, 15). The reasons that lead to inconsistency may arise from different mutation sites, patient ethnicity, and the cohorts’ size. *PIK3CA* exon 9 and exon 20 were located in the helical and kinase domain separately. Mutations at these two domains show a gain of enzymatic function by activating the AKT signaling pathway to induce oncogenic transformation (51). A previous *in vitro* study suspected that the oncogenic mechanisms of these two domains were different. Briefly, mutations in the helical and kinase domain are both required to bind to p85 to induce the gain of function, while the former is required to interact with RAS-GTP simultaneously (52). The different mechanisms could be the reason that leads to the different prognostic effects of *PIK3CA* exon 9 and exon 20.

Chemotherapeutic treatment after surgical resection is a mainstream treatment in stage III CRC patients. 5-fluorouracil is the most widely used drug and is usually combined with other drugs as regimens, such as FOLFOX and XELOX. Recently, more and more CRC patients showed resistance to first-line chemotherapy (21–23). It is urgent to search for biomarkers that could predict chemotherapy resistance in CRC. A few studies suspected that *PIK3CA* mutation is a potential predictive marker in chemotherapy resistance, while the study cohorts were limited and the effect did not focus on stage III CRC patients (24), while patients at stage III with *PIK3CA* mutation were more likely to have disease recurrence or progression.

Nomogram is an effective method to visualize the regression model with various factors, which is valuable in clinical application. Most of the studies focus on constructing nomograms for survival prediction in CRC, including risk factors like TNM stage, age, and tumor location (53, 54). Our study is one of few that tried to construct a nomogram for predicting the recurrence risk of stage III CRC patients (55), and it is the first nomogram that involved *PIK3CA* mutation as a risk factor. The concordance index and the calibration curve indicated that the nomogram we constructed in this study served a well predictive function in clinical practice.

Previous studies indicated that mutation in *PIK3CA* exon 9 did not influence the outcome of anti-EGFR target therapy, while exon 20 mutation might suffer worse outcomes (16). Other studies, however, suspected that *PIK3CA* gene as a whole was a negative biomarker to cetuximab treatment in mCRC patients. The reason that leads to this inconsistency might be that these researches did not exclude *KRAS*, *NRAS*, and *BRAF* mutations during the analysis (17–20). In clinical treatment, bevacizumab treatment is more wildly used for patients with *PIK3CA* mutations. So in our study, only five mCRC patients with *PIK3CA* exon 9 mutations were treated with cetuximab. We found that the DCR in *PIK3CA* exon 9 mutation patients was higher than wild-type patients (100% vs. 88.9%), though the Kaplan-Meier analysis found that the PFS between these two subgroups did not differ significantly (Log-rank test, *p-*value =0.513). With these results, we suggested that *PIK3CA* exon 9 mutation did not affect cetuximab treatment among patients with wild-type *KRAS*, *NRAS*, and *BRAF*. For the low mutation rate, we could not obtain data of exon 20 mutation to cetuximab response. A larger cohort is required to clarify the role of *PIK3CA* in cetuximab resistance in the future.

There are some shortcomings in our study. First of all, though we collected a substantial cohort, patients involved in this study came from a single hospital, so there is a risk of bias in the cohort’s characteristics. Secondly, the concordance index in this nomogram was not very high (0.685). Additional researches were required to build a more reliable predictive model.

## Conclusion

In this study, we uncover the *PIK3CA* mutation profile with a 5763 cohort. We found that 13.4% of Chinese CRC patients carried *PIK3CA* mutation with 58 types of mutants. And 21.4% of mutated patients could be missed if tested with commercial detection kits. We also found that the mutation trend of *PIK3CA* in our cohort remains steady from 2014 to 2018. In survival analysis, we found that *PIK3CA* exon 20 was an adverse biomarker in stage IV CRC patients, and Stage III patients with *PIK3CA* mutation were more likely to have disease recurrence than those with wild-type *PIK3CA*. We also found that *PIK3CA* exon 9 mutations is not a negative biomarker for wild-type *KRAS*, *NRAS*, and *BRAF* mCRC patients in cetuximab treatment.

## Data Availability Statement

The raw data supporting the conclusions of this article will be made available by the authors, without undue reservation.

## Ethics Statement

The studies involving human participants were reviewed and approved by the ethics committee of the Sixth Affiliated Hospital of Sun Yat-sen University. The patients/participants provided their written informed consent to participate in this study.

## Author Contributions

XHF: Conceptualization, methodology, funding acquisition, writing-original draft. HL: methodology, investigation, data curation, writing-original draft. XJF: investigation, data curation, formal analysis. YZ: investigation, data curation; CW: data curation. ZC: investigation, data curation. XT: data curation, investigation. JH: data curation, investigation; YC: software, validation. YH: conceptualization, supervision, writing-review, and editing. All authors contributed to the article and approved the submitted version.

## Funding

This work was supported by the National Key Research and Development Program of China (2017YFC1308800 to Ping Lan), National Natural Science Foundation of China (81971999 to Xin-hui Fu, 81201581 to Jian-ping Wang, 30872488 to Lei Wang), Science and Technology Achievements Transformation Project of Sun Yat-sen University (88000-18843232 to Xin-hui Fu), Young Teacher Training Program of Sun Yat-sen University (14YKPY31 to Xin-hui Fu), Science and Technology Planning Project of Guangdong Province (2012B031800355 to Xin-hui Fu), “985” Project of Sun Yat-sen University (4202037 to Jian-ping Wang), and China Scholarship Council (201706385049 to Xin-hui Fu) and National Key Clinical Discipline. The funder did not influence study design, data collection, and analysis, the decision to publish, or the manuscript preparation.

## Conflict of Interest

The authors declare that the research was conducted in the absence of any commercial or financial relationships that could be construed as a potential conflict of interest.

## Supplementary Material

Supplementary Figure 1 The tendency of *PIK3CA* mutation rates from 2014 to 2018. **(A)** The mutation rate of *PIK3CA* exon 9 in CRC patients is rising, and the estimated value of 2019 is 9.6%. **(B)** The mutation rate of *PIK3CA* exon 20 in CRC patients is slightly increased, and the estimated value in 2019 is 4.7%. **(C)** The mutation rates of *PIK3CA* in CRC patients are increasing, and the estimated value of 2019 is 14.2%.

Supplementary Figure 2 The detection limit of *PIK3CA* exon 9 mutations by the HRM test. The percentage of mutant-type spiked plasmid range from 50% to 0%, and in the 5% test, it still showed a clear bimodal image.

Supplementary Figure 3 The detection limit of *PIK3CA* exon 20 mutations by the HRM test. The percentage of mutant-type spiked plasmid range from 50% to 0%, and in the 5% test, it showed a clear bimodal image.
