# Supplementary figures and images for: The Spectrum, Tendency and Predictive Value of PIK3CA Mutation in Chinese Colorectal Cancer Patients
Source: Front Oncol. 2021 Mar 26;11:595675. doi: 10.3389/fonc.2021.595675 (PMC8032977; doi:10.3389/fonc.2021.595675)

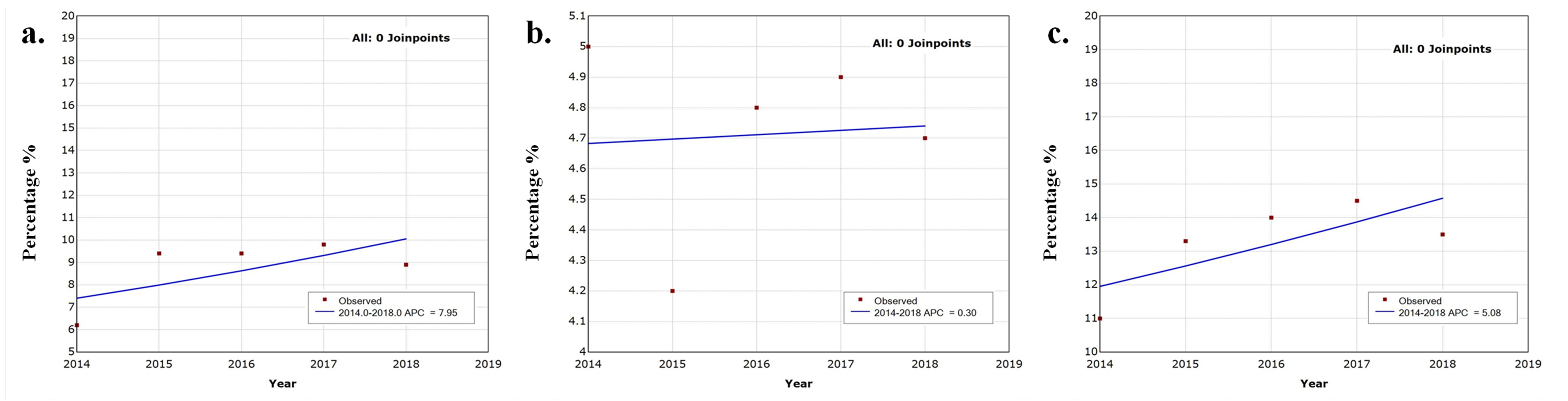

Supplement: Supplementary Figure 1 — The tendency of PIK3CA mutation rates from 2014 to 2018. (A) The mutation rate of PIK3CA exon 9 in CRC patients is rising, and the estimated value of 2019 is 9.6%. (B) The mutation rate of PIK3CA exon 20 in CRC patients is slightly increased, and the estimated value in 2019 is 4.7%. (C) The mutation rates of PIK3CA in CRC patients are increasing, and the estimated value of 2019 is 14.2%. [file Image_1.tif]

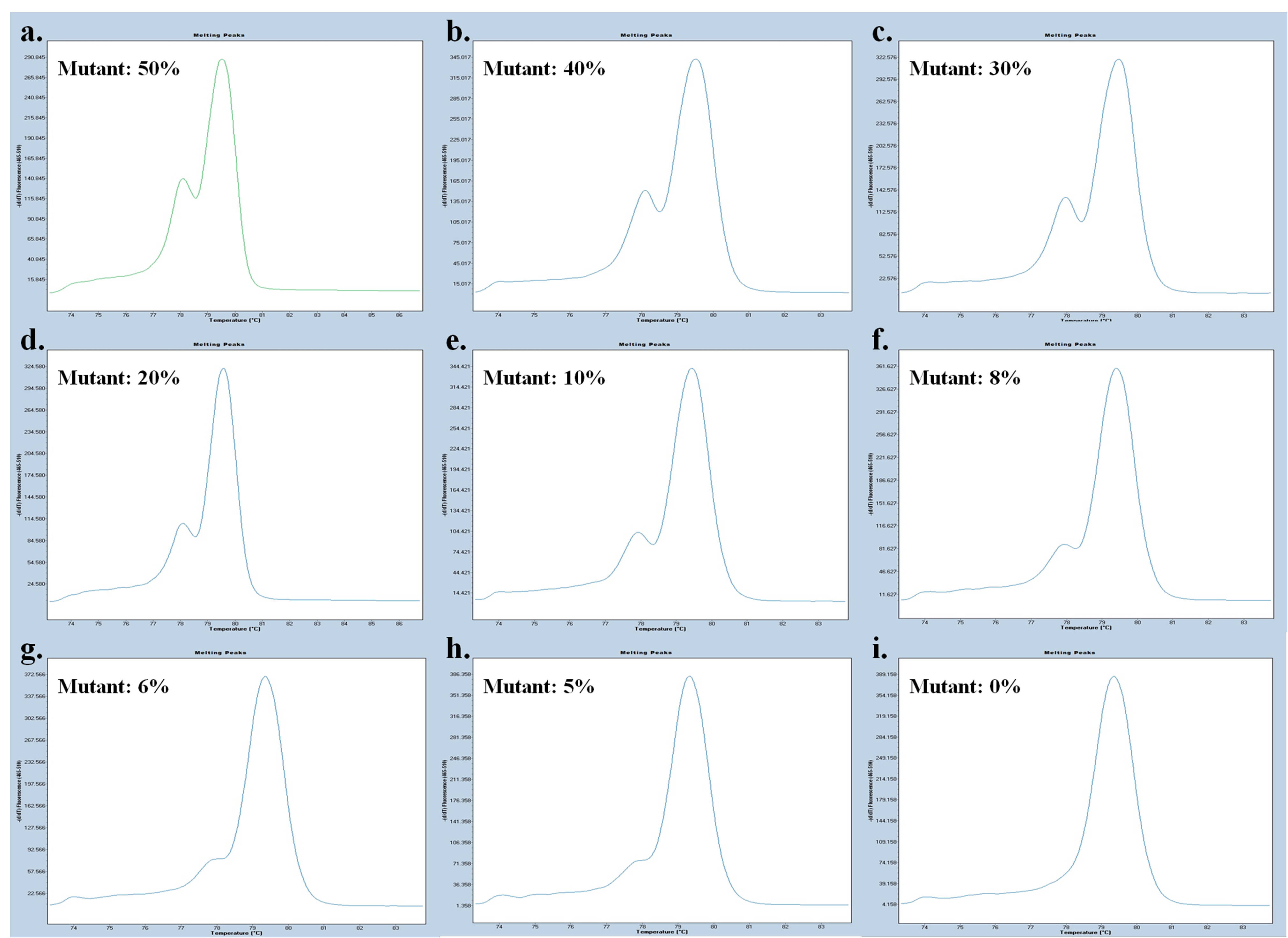

Supplement: Supplementary Figure 2 — The detection limit of PIK3CA exon 9 mutations by the HRM test. The percentage of mutant-type spiked plasmid range from 50% to 0%, and in the 5% test, it still showed a clear bimodal image. [file Image_2.tif]

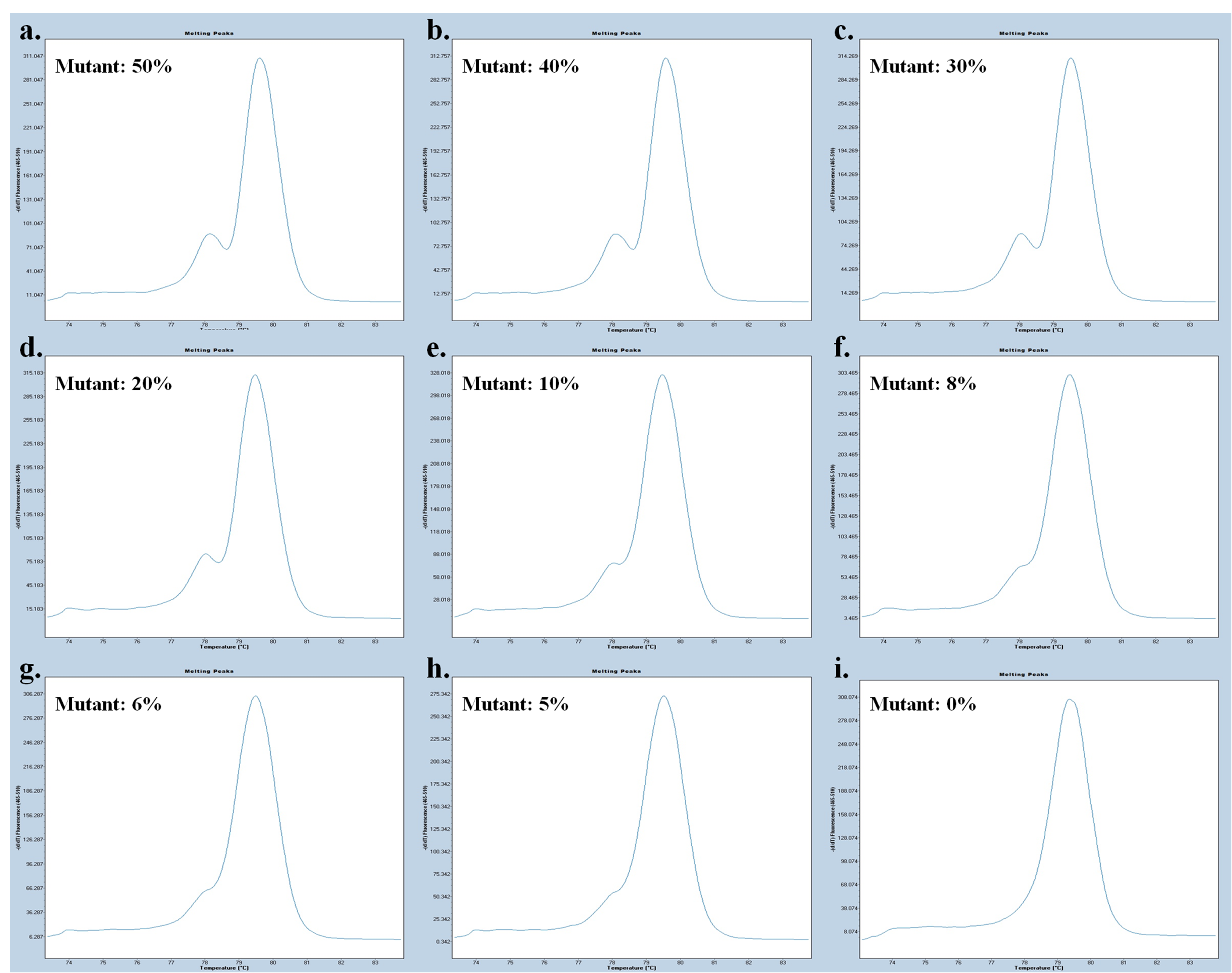

Supplement: Supplementary Figure 3 — The detection limit of PIK3CA exon 20 mutations by the HRM test. The percentage of mutant-type spiked plasmid range from 50% to 0%, and in the 5% test, it showed a clear bimodal image. [file Image_3.tif]
