# Supplementary material for: The Spectrum, Tendency and Predictive Value of PIK3CA Mutation in Chinese Colorectal Cancer Patients
Source: Front Oncol. 2021 Mar 26;11:595675. doi: 10.3389/fonc.2021.595675 (PMC8032977; doi:10.3389/fonc.2021.595675)
Supplement: Supplementary file 4 [file Table_1.docx]

**Table S1.** Associations of *PIK3CA* exon 9 and 20 mutation status with TNM stage

| Characteristics | No. of patients  (n = 3153) | Mutant *PIK3CA*  exon 9 (n = 478) | Wild-type *PIK3CA* exon 9 (n = 2675) | *p* | Mutant *PIK3CA* exon 20(n =253 ) | Wild-type *PIK3CA* exon 20 (n =2900 ) | *p* |
| --- | --- | --- | --- | --- | --- | --- | --- |
| TNM stage |  |  |  | 0.001 |  |  | <0.001 |
| Stage I | 344 (10.9%) | 44 (12.8%) | 300 (87.2%) |  | 19 (5.5%) | 325 (94.5%) |  |
| Stage II | 1187 (37.6%) | 217 (18.3%) | 970 (81.7%) |  | 135 (11.4%) | 1052 (88.6%) |  |
| Stage III | 1101 (34.9%) | 136 (12.4%) | 965 (87.6%) |  | 65 (5.9%) | 1036 (94.1%) |  |
| Stage IV | 521 (16.5%) | 81 (15.5%) | 440 (84.5%) |  | 34 (6.5%) | 487 (93.5%) |  |
