# Supplementary material for: The Spectrum, Tendency and Predictive Value of PIK3CA Mutation in Chinese Colorectal Cancer Patients
Source: Front Oncol. 2021 Mar 26;11:595675. doi: 10.3389/fonc.2021.595675 (PMC8032977; doi:10.3389/fonc.2021.595675)
Supplement: Supplementary file 5 [file Table_2.docx]

**Table S2.** Logistic regression model associations between clinicopathologic characteristics and *PIK3CA* exon 9 and 20 mutations

| **Factor** | **Mutant *PIK3CA* exon 9** | | | | **Mutant *PIK3CA* exon 20** | | | |
| --- | --- | --- | --- | --- | --- | --- | --- | --- |
|  | **Univariate analysis** | | **Multivariate analysis** | | **Univariate analysis** | | **Multivariate analysis** | |
|  | **OR (95% CIs)** | ***p*** | **OR (95% CIs)** | ***p*** | **OR (95% CIs)** | ***p*** | **OR (95% CIs)** | ***p*** |
| Right colon (*vs.* others) | 1.86 (1.53-2.26) | <0.001 | 2.01 (1.63-2.48) | <0.001 | 2.72 (2.12-3.50) | < 0.001 | 2.71 (2.10-3.50) | <0.001 |
| Poor differentiation (*vs.* others) | 0.51 (0.32-0.81) | 0.004 | 0.46 (0.29-0.74) | 0.001 | 1.07 (0.67-1.72) | 0.766 | 0.88 (0.55-1.42) | 0.595 |
| Age < 50 years (*vs*. ≥50 years) | 0.99 (0.80-1.23) | 0.929 | 1.10 (0.88-1.38) | 0.418 | 1.70 (1.31-2.21) | < 0.001 | 1.68 (1.29-2.19) | <0.001 |
| Stage II (*vs.* others)* | 1.46 (1.20-1.78) | <0.001 | 1.40 (1.15-1.70) | <0.001 | 2.01 (1.55-2.60) | < 0.001 | 1.94 (1.50-2.53) | <0.001 |

CIs = confidence interval; OR = odds ratio.

Stage II (*vs.* others)*: only 3153 patients were involved for analyses.
