# Supplementary material for: The Spectrum, Tendency and Predictive Value of PIK3CA Mutation in Chinese Colorectal Cancer Patients
Source: Front Oncol. 2021 Mar 26;11:595675. doi: 10.3389/fonc.2021.595675 (PMC8032977; doi:10.3389/fonc.2021.595675)
Supplement: Supplementary file 6 [file Table_3.docx]

**Table S3.** The mutation rates of *PIK3CA* from 2014-2018

| *PIK3CA* mutation | 2014 | 2015 | 2016 | 2017 | 2018 |
| --- | --- | --- | --- | --- | --- |
| Exon 9 | 6.2% (48/780) | 9.4% (87/928) | 9.4% (93/987) | 9.8% (138/1413) | 8.9% (145/1625) |
| Exon 20 | 5.0% (39/780) | 4.2% (39/928) | 4.8% (47/987) | 4.9% (69/1413) | 4.7% (76/1625) |
| Total | 11.0% (86/780) | 13.3% (123/928) | 14.0% (138/987) | 14.5% (205/1413) | 13.5% (219/1625) |
