# Supplementary material for: The Spectrum, Tendency and Predictive Value of PIK3CA Mutation in Chinese Colorectal Cancer Patients
Source: Front Oncol. 2021 Mar 26;11:595675. doi: 10.3389/fonc.2021.595675 (PMC8032977; doi:10.3389/fonc.2021.595675)
Supplement: Supplementary file 7 [file Table_4.docx]

**Table S4.** Associations of *PIK3CA* exon 9 and 20 mutation status with clinicopathologic characteristics in 1946 cohort

| Characteristics | No. of patients  (n = 1946) | Mutant *PIK3CA* exon 9 (n = 447) | Wild-type *PIK3CA* exon 9 (n = 1499) | *p* | Mutant *PIK3CA* exon 20 (n = 238) | Wild-type *PIK3CA* exon 20 (n = 1708) | *p* |
| --- | --- | --- | --- | --- | --- | --- | --- |
| Gender |  |  |  | 0.955 |  |  | 0.149 |
| Male | 1246 (64.0%) | 287 (23.0%) | 959 (77.0%) |  | 142 (11.4%) | 1104 (88.6%) |  |
| Female | 700 (36.0%) | 160 (22.9%) | 540 (77.1%) |  | 96 (13.7%) | 604 (86.3%) |  |
| Age, years |  |  |  | 0.127^1^ |  |  | 0.001^1^ |
| Mean (SD) | 58.5 (12.9) | 59.3 (13.8) | 58.3 (12.6) |  | 55.8 (14.1) | 58.9 (12.7) |  |
| Median | 60.0 | 60.0 | 59.0 |  | 58.0 | 60.0 |  |
| Range | 17.0-95.0 | 23.0-95.0 | 17.0-90.0 |  | 18.0-89.0 | 17.0-95.0 |  |
| Age, years |  |  |  | 0.015 |  |  | 0.001 |
| <45 | 273 (14.0%) | 66 (24.2%) | 207 (75.8%) |  | 52 (19.0%) | 221 (81.0%) |  |
| 45-49 | 199 (10.2%) | 35 (17.6%) | 164 (82.4%) |  | 30 (15.1%) | 169 (84.9%) |  |
| 50-75 | 1290 (66.3%) | 286 (22.4%) | 1001 (77.6%) |  | 137 (10.6%) | 1153 (89.4%) |  |
| >75 | 184 (9.5%) | 57 (31.0%) | 127 (69.0%) |  | 19 (10.3%) | 165 (89.7%) |  |
| Tumor site |  |  |  | 0.086 |  |  | 0.006 |
| Rectum | 948 (48.7%) | 226 (23.8%) | 722 (76.2%) |  | 104 (17.7%) | 844 (89.0%) |  |
| Left colon* | 693 (35.6%) | 141 (20.3%) | 552 (79.7%) |  | 80 (11.5%) | 613 (88.5%) |  |
| Right colon* | 305 (15.7%) | 80 (26.2%) | 225 (73.8%) |  | 54 (11.0%) | 251 (82.3%) |  |
| Differentiation of tubular adenocarcinoma |  |  |  | 0.001 |  |  | 0.293 |
| Well | 286 (16.4) | 80 (28.0%) | 206 (72.0%) |  | 38 (13.3%) | 248 (86.7%) |  |
| Moderate | 1384 (71.1%) | 307 (22.2%) | 1077 (77.8%) |  | 153 (11.1%) | 1231 (88.9%) |  |
| Poor | 129 (6.6%) | 15 (11.6%) | 114 (88.4%) |  | 19 (14.7%) | 110 (85.3%) |  |
| Nontubular adenocarcinoma | 148 | 45 | 103 |  | 29 | 119 |  |
| TNM stage |  |  |  | 0.013 |  |  | <0.001 |
| Stage I | 239 (12.3%) | 42 (17.6%) | 197 (82.4%) |  | 17 (7.1%) | 222 (92.9%) |  |
| Stage II | 802 (41.2%) | 206 (25.7%) | 596 (74.3%) |  | 128 (16.0%) | 674 (84.0%) |  |
| Stage III | 619 (31.8%) | 126 (20.4%) | 493 (79.6%) |  | 62 (10.0%) | 557 (90.0%) |  |
| Stage IV | 286 (14.7%) | 73 (25.5%) | 213 (74.5%) |  | 31 (10.8%) | 255 (89.2%) |  |
| *PIK3CA* exon 20 status |  |  |  | <0.001 |  |  | -- |
| Wild-type | 1708 (87.8%) | 438 (25.6%) | 1270 (74.4%) |  |  |  |  |
| Mutant | 238 (12.2%) | 9 (3.8%) | 229 (96.2%) |  |  |  |  |
| *PIK3CA* exon 9 status |  |  |  | -- |  |  | <0.001 |
| Wild-type | 1499 (77.0%) |  |  |  | 229 (15.3%) | 1270 (84.7%) |  |
| Mutant | 447 (23.0%) |  |  |  | 9 (2.0%) | 438 (98.0%) |  |
| *KRAS* exon 2 status |  |  |  | <0.001 |  |  | <0.001 |
| Wild-type | 1539 (79.1%) | 277 (18.0%) | 1262 (82.0%) |  | 125 (8.1%) | 1414 (91.9%) |  |
| Mutant | 407 (20.9%) | 170 (41.8%) | 237 (58.2) |  | 113 (27.8%) | 294 (72.2%) |  |
| *BRAF^V600E^* status |  |  |  | 0.128 |  |  | 0.009 |
| Wild-type | 1915 (98.4%) | 436 (22.8%) | 1479 (77.2%) |  | 229 (12.0%) | 1686 (88.0%) |  |
| Mutant | 31 (1.6%) | 11 (35.5%) | 20 (64.5%) |  | 9 (29.0%) | 20 (71.0%) |  |

*Left colon: descending colon, sigmoid colon, and rectosigmoid; Right colon: cecum, ascending colon and transverse colon.

Spearman Chi-square test.

^1^Mann-Whitney U test.
