# Supplementary material for: The Spectrum, Tendency and Predictive Value of PIK3CA Mutation in Chinese Colorectal Cancer Patients
Source: Front Oncol. 2021 Mar 26;11:595675. doi: 10.3389/fonc.2021.595675 (PMC8032977; doi:10.3389/fonc.2021.595675)
Supplement: Supplementary file 8 [file Table_5.docx]

**Table S5.** Associations of *PIK3CA* exon 9 and 20 mutation status with clinicopathologic characteristics in 377 cohort

| Characteristics | No. of patients  (n = 377) | Mutant *PIK3CA* exon 9 (n = 68) | Wild-type *PIK3CA* exon 9 (n = 309) | *p* | Mutant *PIK3CA* exon 20 (n = 33) | Wild-type *PIK3CA* exon 20 (n = 344) | *p* |
| --- | --- | --- | --- | --- | --- | --- | --- |
| Gender |  |  |  | 0.221 |  |  | 0.042 |
| Male | 221 (58.6%) | 35 (15.8%) | 186 (84.2%) |  | 25 (11.3%) | 196 (88.7%) |  |
| Female | 156 (41.4%) | 33 (21.2%) | 123 (78.8%) |  | 8 (5.1%) | 148 (94.9%) |  |
| Age, years |  |  |  | 0.486^1^ |  |  | 0.024^1^ |
| Mean (SD) | 54.4 (12.5) | 53.7 (12.9) | 54.6 (12.4) |  | 49.4 (13.0) | 54.9 (12.3) |  |
| Median | 57.0 | 55.0 | 57.0 |  | 52.0 | 57.0 |  |
| Range | 19.0-83.0 | 25.0-78.0 | 19.0-83.0 |  | 23.0-68.0 | 19.0-83.0 |  |
| Age, years |  |  |  | 0.219 |  |  | 0.222 |
| <45 | 82 (21.8%) | 19 (23.2%) | 63 (76.8%) |  | 12 (14.6%) | 70 (85.4%) |  |
| 45-49 | 42 (11.1%) | 7 (16.7%) | 35 (83.3%) |  | 3 (7.1%) | 39 (92.9%) |  |
| 50-75 | 245 (65.0%) | 39 (15.9%) | 206 (84.1%) |  | 18 (7.3%) | 227 (92.7%) |  |
| >75 | 8 (2.1%) | 3 (37.5%) | 5 (62.5%) |  | 0 (0%) | 8 (100%) |  |
| Tumor site |  |  |  | 0.144 |  |  | 0.087 |
| Rectum | 118 (31.3%) | 28 (23.7%) | 90 (76.3%) |  | 12 (10.2%) | 106 (89.8%) |  |
| Left colon | 156 (41.4%) | 23 (14.7%) | 133 (85.3%) |  | 8 (5.1%) | 148 (94.9%) |  |
| Right colon | 103 (27.3%) | 17 (16.5%) | 86 (83.5%) |  | 13 (12.6%) | 90 (87.4%) |  |
| Differentiation of tubular adenocarcinoma |  |  |  | 0.677 |  |  | 0.191 |
| Well | 28 (7.4%) | 6 (21.4%) | 22 (78.6%) |  | 5 (17.9%) | 23 (82.1%) |  |
| Moderate | 280 (74.3%) | 50 (17.9%) | 230 (82.1%) |  | 23 (8.2%) | 257 (91.8%) |  |
| Poor | 32 (8.5%) | 4 (12.5%) | 28 (87.5%) |  | 2 (6.2%) | 30 (93.8%) |  |
| Nontubular adenocarcinoma | 37 | 8 | 29 |  | 3 | 34 |  |
| *PIK3CA* exon 20 status |  |  |  | 0.016^2^ |  |  | -- |
| Wild-type | 344 (91.2%) | 67 (19.5%) | 277 (80.5%) |  |  |  |  |
| Mutant | 33 (8.8%) | 1 (3.0%) | 32 (97.0%) |  |  |  |  |
| *PIK3CA* exon 9 status |  |  |  | -- |  |  | 0.016^2^ |
| Wild-type | 309 (82.0%) |  |  |  | 32 (10.4%) | 277 (89.6%) |  |
| Mutant | 68 (18.0%) |  |  |  | 1 (1.5%) | 67 (98.5%) |  |
| *KRAS* exon 2 status |  |  |  | <0.001 |  |  | 0.854 |
| Wild-type | 217 (57.6%) | 24 (11.1%) | 193 (88.9%) |  | 18 (8.3%) | 199 (91.7%) |  |
| Mutant | 160 (42.4%) | 44 (27.5%) | 116 (72.5%) |  | 15 (9.4%) | 145 (90.6%) |  |
| *BRAF^V600E^* status |  |  |  | 0.325^2^ |  |  | 0.631^2^ |
| Wild-type | 362 (96.0%) | 64 (17.7%) | 298 (82.3%) |  | 31 (8.6%) | 331 (91.4%) |  |
| Mutant | 15 (4.0%) | 4 (26.7%) | 11 (73.3%) |  | 2 (13.3%) | 13 (86.7%) |  |

*Left colon: descending colon, sigmoid colon, and rectosigmoid; Right colon: cecum, ascending colon and transverse colon.

Spearman Chi-square test.

^1^Mann-Whitney U test.

^2^Fisher’s exact test
